# *Centranthus ruber* (L.) DC. and *Tropaeolum majus* L.: Phytochemical Profile, In Vitro Anti-Denaturation Effects and Lipase Inhibitory Activity of Two Ornamental Plants Traditionally Used as Herbal Remedies

**DOI:** 10.3390/molecules28010032

**Published:** 2022-12-21

**Authors:** Vincenzo Musolino, Mariangela Marrelli, Maria Rosaria Perri, Martina Palermo, Micaela Gliozzi, Vincenzo Mollace, Filomena Conforti

**Affiliations:** 1 Laboratory of Pharmaceutical Biology, Department of Health Sciences, Institute of Research for Food Safety and Health (IRC-FSH), University Magna Græcia of Catanzaro, 88100 Catanzaro, Italy; 2 Department of Pharmacy, Health and Nutritional Sciences, University of Calabria, Rende, 87036 Cosenza, Italy; 3 Department of Health Sciences, Institute of Research for Food Safety and Health (IRC-FSH), University Magna Græcia of Catanzaro, 88100 Catanzaro, Italy

**Keywords:** anti-denaturation activity, antioxidant, bovine serum albumin, garden nasturtium, Indian cress, nitric oxide, pancreatic lipase, *Valeriana rubra* L.

## Abstract

Ornamental plants often gain relevance not only for their decorative use, but also as a source of phytochemicals with interesting healing properties. Herein, spontaneous *Centranthus ruber* (L.) DC. and *Tropaeolum majus* L., mainly used as ornamental species but also traditionally consumed and used in popular medicine, were investigated. The aerial parts were extracted with methanol trough maceration, and resultant crude extracts were partitioned using solvents with increasing polarity. As previous studies mostly dealt with the phenolic content of these species, the phytochemical investigation mainly focused on nonpolar constituents, detected with GC-MS. The total phenolic and flavonoid content was also verified, and HPTLC analyses were performed. In order to explore the potential antiarthritic and anti-obesity properties, extracts and their fractions were evaluated for their anti-denaturation effects, with the use of the BSA assay, and for their ability to inhibit pancreatic lipase. The antioxidant properties and the inhibitory activity on the NO production were verified, as well. Almost all the extracts and fractions demonstrated good inhibitory effects on NO production. The *n*-hexane and dichloromethane fractions from *T. majus*, as well as the *n*-hexane fraction from *C. ruber*, were effective in protecting the protein from heat-induced denaturation (IC_50_ = 154.0 ± 1.9, 270.8 ± 2.3 and 450.1 ± 15.5 μg/mL, respectively). The dichloromethane fractions from both raw extracts were also effective in inhibiting pancreatic lipase, with IC_50_ values equal to 2.23 ± 0.02 mg/mL (for *C. ruber* sample), and 2.05 ± 0.02 mg/mL (*T. majus)*. Obtained results support the traditional use of these species for their beneficial health properties and suggest that investigated plant species could be potential sources of novel antiarthritic and anti-obesity agents.

## 1. Introduction

Ornamental plant species often constitute a source of natural compounds with beneficial health-promoting properties, widely used in the food industry and cosmetics because of both their beneficial properties and fragrance [1]. 

Different ornamental species, e.g., hibiscus, marigold, calendula, aloe vera and agave, even if being used as indoor plants, are also cultivated for their medicinal use because of their interesting content in bioactive compounds such as phenolics, essential oils and other secondary metabolites [2]. Nevertheless, the flowers from some of these plants, such as dandelion and Japanese honeysuckle, are edible and are often used for making sweet-dishes or beverages [3]. Mlcek and Rop described the edible flowers from ornamental plant species as a new source of nutraceuticals, because of their nutritional and chemoprotective value, mainly linked to their phenolic content and their radical scavenging activity [4].

According to these observations, a number of recent studies focus on the in vitro properties of plant species mostly used as ornamental [5,6,7].

In our previous studies focusing on the search for bioactive phytochemicals from Mediterranean wild edible plants, we explored the biological properties of different species from southern Italy. We paid particular attention towards phytoalimurgy, the branch of botany focused on the rediscovery and the study of wild plants for their nutritional value [8] and towards those species traditionally used for medicinal purpose in our popular medicine. Some of these species, such as *Capparis sicula* Veill. spp. *sicula*, *Borago officinalis* L., *Clematis vitalba* L. and *Portulaca oleracea* L., showed very interesting inhibitory properties on digestive enzymes [9,10,11]. Other species traditionally consumed in southern Italy demonstrated antioxidant and/or anti-inflammatory potential, such as *Rubus caesius* L., *Mentha aquatica* L. and *Echium vulgare* L. [12,13,14,15], and also antiarthritic potential, as *Daucus carota* L. [16]. 

Given all these premises, as part of our studies focusing on the beneficial health properties of plant species which are part of the flora of southern Italy, and based on the bibliographic search we carried out during these years, here, we wanted to focus our work on the investigation of the phytochemical content and the healing properties of two ornamental plants collected in Calabria: *Centranthus ruber* (L.) DC. and *Tropaeolum majus* L. We decided to study these species, as they are both not only ornamental plants but also have a popular traditional culinary use in Italy, which induced us to explore their nutraceutical value, and a traditional medicinal use, which motivated us to investigate some biological properties which, to the best of our knowledge, have never been explored before. 

*Centranthus ruber* (L.) DC. (synonym of *Valeriana rubra* L.) is commonly known as red valerian. Even if it has long been regarded as belonging to the Valerianaceae Batsch family, it has been more recently included in the Caprifoliaceae Juss. [17,18,19]. Specifically, this plant is now considered a member of Valerianaceae, a sub-family of Caprifoliaceae which comprises about 315 species of annual and perennial herbs [20]. *Centranthus* genus comprises about 11 species with circum-Mediterranean and European distribution, even if some species have been introduced or naturalized in other parts of the world [19].

*C. ruber* (L.) DC. is endemic of the Mediterranean and Balkan countries, southern France and Sardinia, and it is naturalized in different countries such as USA, Australia and New Zealand [21].

Due to its ability to easily regrow from its roots, Holmes and coworkers investigated the invasive potential of *C. ruber* through the evaluation of the persistence and spread of this species across the urban gradient [21]. 

In Italy, the boiled leaves and shoots from this plant are traditionally used as depurative, as well as in food, for the preparation of a vegetable pie or as a side dish [20]. The leaves are used as an antispasmodic and eaten raw in salads [22]. 

As other plants belonging to Caprifoliaceae, *C. ruber* L. also contains the cyclopentan-c-pyran monoterpenoids called valepotriates, a name that derives from the contraction of valeriana-epoxy-triesters. These iridoids have been demonstrated to induce mutagenic and carcinogenic properties [23]. The most common and employed plant belonging to Valerianaceae, *Valeriana officinalis* L., has been in use for a long time for its antispasmodic, relaxing and sedative properties, mainly attributed to the presence of valepotriates and borneol derivatives [20]. 

*Tropaeolum majus* L., commonly known as garden nasturtium or Indian cress, belongs to the Tropaeolaceae family. This plant is native to Central and South America, and it was introduced in Europe in the XVI century, where it is mainly cultivated as a decorative plant [24]. 

The flowers of this plant also have a culinary use, as they are added to salads [25], and this species has been also traditionally used in wound-healing and as a disinfectant and expectorant [26]. Gomes and coworkers demonstrated the absence of subchronic toxicity due to oral treatment with the hydroalcoholic extract of *T. majus* in vivo [27]. 

The presence of flavonoids and other phenolic compounds and the antioxidant activity have been demonstrated [24,26,28,29,30]. Moreover, some other biological properties have been described for the extracts from this species. Gasparotto Junior and colleagues reported the in vivo antihypertensive effects of the hydroalcoholic extract from the leaves of the plant [31]. The diuretic effects have also been explored [32], as well as the antibacterial activity [25] and antihypertensive [31] and protective cardiorenal effects [33]. 

The essential oil from the flowers and leaves of the plant also demonstrated antimicrobial and anti-inflammatory activities [34].

In the present study, we aimed to further investigate the phytochemical and biological properties of these two very interesting ornamental species. As previous research on these plants mostly dealt with their phenolic content, the chemical characterization mainly focused on the nonpolar constituents, namely fatty acids, terpenes and phytosterols, detected with gas chromatography–mass spectrometry (GC–MS). The total phenolic and flavonoid content was verified as well, and a rapid screening of some already known polar compounds was also carried out by means of high-performance thin-layer chromatography (HPTLC). 

In order to evaluate the potential antiarthritic activity of *C. ruber* and *T. majus*, the extracts and their fractions were investigated for their anti-denaturation effects with the use of the bovine serum albumin (BSA) assay. The antioxidant and the inhibitory activity on the nitric oxide (NO) production were verified as well. Among the wide range of cellular and physiological processes in which this signaling molecule is involved, NO also plays a role in the progression of osteoarthritis: an increase in its level is considered to be involved in the activation of TNFα and IL-1β, and it is related to cartilage chondrocyte apoptosis [35].

Furthermore, given the traditional culinary use of both these species, we also aimed to estimate their potential nutraceutical value, focusing our attention on the potential health benefits in the treatment of obesity. To this end, extracts and their fractions were also evaluated for their inhibitory properties on pancreatic lipase, a key enzyme for dietary fats absorption.

## 2. Results and Discussion

### 2.1. Extraction Yields

The aerial parts from both *C. ruber* and *T. majus* collected in southern Italy were extracted with methanol through maceration (48 h × 3 times, plant to solvent ratio 1:10 g/mL). The yields obtained for the two raw extracts were 13.5% and 8.0%. 

Moreover, both crude extracts were subjected to sequential fractionations using solvents with increasing polarity, namely *n*-hexane (with yields equal to 0.8% and 1.3% referred to dry plant material for *C. ruber* and *T. majus*, respectively), dichloromethane (1.3% and 0.9%) and ethyl acetate (0.7% and 0.4%). The yields of the aqueous residues were 10.7% for *C. ruber* and 5.4% for *T. majus*.

### 2.2. Phytochemical Analysis

GC-MS analyses allowed the identification of the chemical compounds present in the nonpolar fractions of the two raw extracts (*n*-hexane and dichloromethane). As reported in Table 1, both the *n*-hexane fractions showed a high number of fatty acids. Overall, 16 compounds belonging to this class were identified, with palmitic acid being the most abundant (6.87% and 5.08% for *C. ruber* and *T. majus*, respectively). Furthermore, behenic acid was detected at percentages above 2% and 9-octadecenoic acid and stearic acid at percentages above 1%. The two essential fatty acids, linoleic and α-linolenic, were detected in *T. majus* only. Four terpenes were also identified: the monoterpene lactone dihydroactinidioide and the isoprenoid ketone phytone (hexahydrofarnesyl acetone), which were detected in both samples, the monoterpenoid 2,6-di-tert-butyl-1,4-benzoquinone (present in *C. ruber*) and the diterpene neophytadiene *(T. majus*). The two samples presented a different composition in regard to the phytosterol content: campesterol and stigmasterol were identified in *C. ruber*, while β-sitosterol and tremulone were detected in the *T. majus n*-hexane fraction. Moreover, β-tocopherol was detected in *T. majus* only.

The chemical constituents identified in the two dichloromethane fractions are reported in Table 2. The alpha-hydroxy acid mandelic acid was the most abundant compound in the *T. majus* sample (6.63%), followed by the monoterpene lactone loliolide (2.21%). Both of these compounds were not detected in the *C. ruber* dichloromethane fraction, whose main components were benzoic acid (1.10%) and ethylmethylmaleimide (0.51%). 

**Figure 1:**
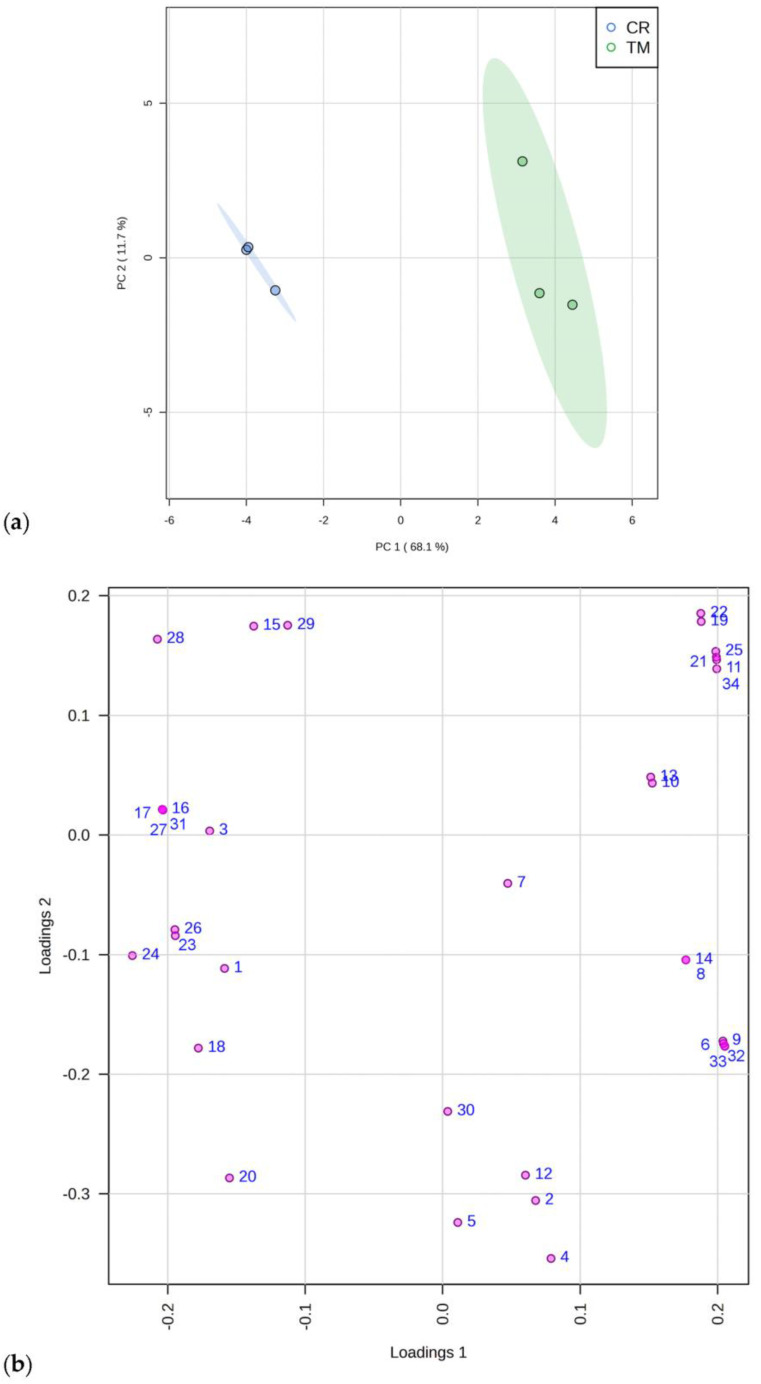
Principal component analysis of 34 constituents identified with GC-MS in *C. ruber* (L.). DC. and *T. majus* L. samples. (**a**) Scores plot and (**b**) loadings plot. CR, *C. ruber*; TM, *T. majus*. The serial numbers of phytochemicals are consistent with peak numbering in Table 1 and Table 2: 1, isolauric acid; 2, lauric acid; 3, azelaic acid; 4, myristic acid; 5, pentadecanoic acid; 6, 7,10,13-hexadecatrienoic acid; 7, palmitic acid; 8, margaric acid; 9, α-linolenic acid; 10, linoleic acid; 11, 9-octadecenoic acid; 12, stearic acid; 13, arachidic acid; 14, heneicosanoic acid; 15, behenic acid; 16, tricosylic acid; 17, 2,6-di-tert-butyl-1,4-benzoquinone; 18, dihydroactinidiolide; 19, neophytadiene; 20, phytone; 21, campesterol; 22, stigmasterol; 23, β-sitosterol; 24, tremulone; 25, β-tocopherol; 26, 3-cresol; 27, benzoic acid; 28, ethylmethylmaleimide; 29, cinnamic acid; 30, isoeugenol; 31, 2,6-bis(tert-butyl)phenol; 32, mandelic acid; 33, vanillic acid; 34, loliolide.

A Principal Component Analysis (PCA), which evidenced these data patterns, is reported in Figure 1, reporting the scores (a) and loading plots (b) by considering the first and the second principal components (PC-1 vs. PC2), with a total explained variance of 79.8%. The data matrix consisted of 6 samples (3 determinations for each plant species) and 39 descriptor variables. 

Our results are in agreement with those of Tran and coworkers, who reported the presence of mandelic acid in *T. majus* aqueous extract [36].

As regards the content in polar compounds of the two investigated species, we firstly performed two colorimetric assays in order to quantify the total phenolic and total flavonoid content. *T. majus* showed a higher amount of phenolics compared to the second species, with 140.2 ± 1.4 and 119.7 ± 0.6 mg/g, expressed as chlorogenic acid equivalents per g of plant extract. A similar trend was observed for the total flavonoid content, with values of 13.24 ± 0.02 and 10.04 ± 0.01 mg/g of extract for *T. majus* and *C. ruber*, respectively.

As previous works on *C. ruber* and *T. majus* phytochemical profiles mainly dealt with phenolic compounds, the chemical characterization was here mainly focused on the nonpolar constituents. However, in order to identify some of the most common phenolic compounds, the two polar fractions (ethyl acetate samples) of both plant extracts were analyzed using HPTLC, a practical and rapid technique for the chemical characterization of complex mixtures of natural substances [37,38]. 

**Figure 2:**
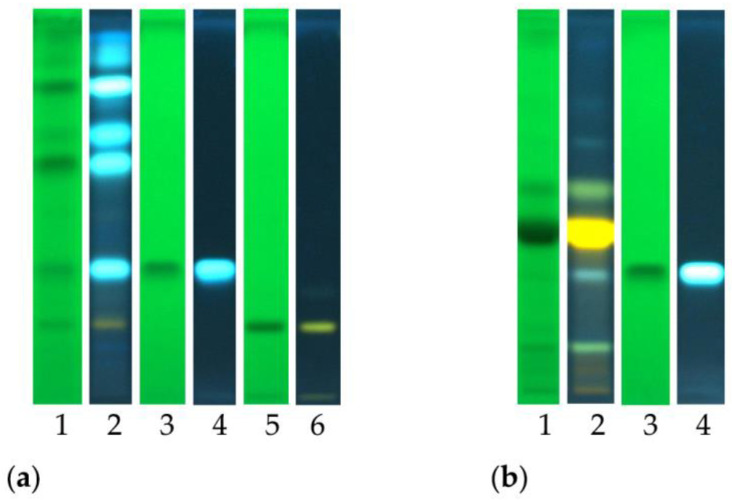
High-performance thin-layer chromatography (HPTLC) fingerprints of (**a**) *C. ruber* (L.) DC. and (**b**) *T. majus* L. ethyl acetate fractions. Mobile phase: Ethyl acetate/dichloromethane/acetic acid/formic acid/water (100:25:10:10:11, v/v/v/v/v). Tracks: 1, sample visualized at 254 nm; 2, sample at 366 nm and NP derivatization; 3, chlorogenic acid ad 254 nm; 4, chlorogenic acid at 366 nm; 5, rutin at 254 nm; 6, rutin at 366 nm.

The analyses performed allowed us to tentatively detect the presence of chlorogenic acid and the flavonoid glycoside rutin. The obtained results are reported in Figure 2, which reports the chemical fingerprinting of analyzed samples and the co-chromatography with reference compounds. Chlorogenic acid was identified in the ethyl acetate fraction of both crude extracts, as indicated by the typical blue spot. The flavonoid glycoside rutin was detected in the AcOEt fraction of *C. ruber* crude extract, and it is recognizable as a yellow spot after post chromatographic exposure to Natural Product (NP) reagent. 

**Figure 3:**
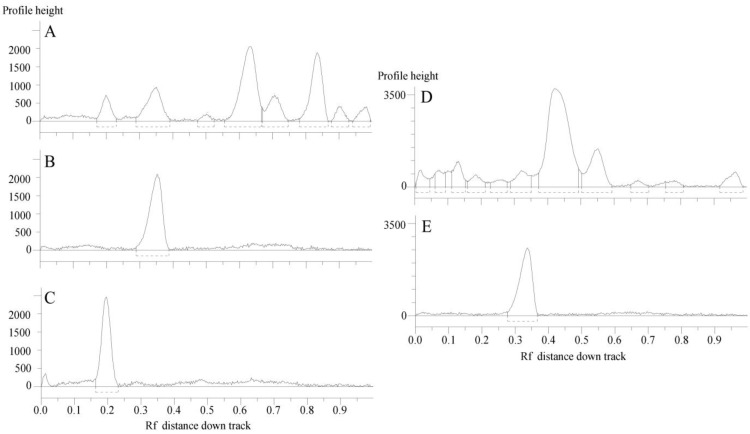
HPTLC chromatograms of the ethyl acetate fractions from crude extracts and utilized standards. (**A**) *C. ruber* (L.) CD.; (**B**) chlorogenic acid (Rf = 0.35); (**C**) rutin (Rf = 0.20); (**D**) *T. majus* L., (**E**) rutin. Mobile phase: Ethyl acetate/dichloromethane/acetic acid/formic acid/water (100:25:10:10:11, v/v/v/v/v).

Figure 3 reports the HPTLC chromatographic profile of *C. ruber* and *T. majus* ethyl acetate fractions and utilized reference standards, chlorogenic acid and rutin.

### 2.3. In Vitro Antioxidant Potential

The in vitro antioxidant potential of *C. ruber* and *T. majus* extracts and their fractions was verified by means of two colorimetric assays, the DPPH and the β-carotene bleaching test. Both the raw extracts showed radical scavenging potency (Table 3), with the *T. majus* sample being more active (IC_50_ = 53.34 ± 0.34 μg/mL) than *C. ruber* (IC_50_ = 79.86 ± 1.27 μg/mL, *p* < 0.05, Bonferroni post-hoc test). The fractions were effective as well, with the only exception being the *n*-hexane samples. The ethyl acetate fraction of *T. majus* showed the best activity, with an IC_50_ value equal to 14.08 ± 0.29 μg/mL. A good radical scavenging acitvity was also observed for the AcOEt fraction of *C. ruber*, even if to a minor extent (IC_50_ value equal to 84.22 ± 0.48 μg/mL).

Investigated crude extracts were also effective in protecting linoleic acid from peroxidation, as assessed in the β-carotene bleaching test (Table 3). No statistically significant differences were detected among the respective IC_50_ values after 30 min of incubation (38.15 ± 1.34 and 39.57 ± 0.49 μg/mL). The *C. ruber* raw sample was also effective after 60 min (IC_50_ = 62.58 ± 2.51 μg/mL). The best antioxidant activity was demonstrated by the *C. ruber* ethyl acetate fraction, with an IC_50_ value equal to 28.17 ± 0.49 μg/mL after 30 min of incubation. 

Our results are in agreement with previous studies dealing with the biological properties of these two species, mainly focusing on the antioxidant potency. Vanzani and coworkers reported the antioxidant activity of the hydroalcoholic extract from *C. ruber* collected in northern Italy, which was assessed with the ferric reducing antioxidant power (FRAP assay) and the measurement of the peroxyl radical scavenging activity [22]. Unlike *C. ruber*, the antioxidant potential of *T. majus* has already been deeply explored. The extract of the flowers of the plant from Colombia showed a good ABTS and DPPH radical scavenging activity [26]. Bazylko and coworkers reported a strong scavenging activity against reactive nitrogen species for both the aqueous and hydroethanolic extracts [30].

### 2.4. In Vitro Nitric Oxide (NO) Production Inhibition

As one of the aims of our study was to assess the antiarthritic potential of *C. ruber* and *T. majus* extracts, we wanted to evaluate the inhibitory properties on the production of nitric oxide. This pro-inflammatory mediator plays a role in the progression of osteoarthritis as an increase in its level, produced by inducible nitric oxide synthase (iNOS), is a catabolic element in the activation of TNFα and IL-1β, and it is related to cartilage chondrocyte apoptosis [35].

The inhibitory properties on NO production were verified in LPS-stimulated RAW 264.7 macrophages treated with different concentrations of investigated extracts and their fractions. An indirect determination of nitric oxide was carried out through the spectrophotometric measurement of its stable end-products, nitrite and nitrate, using the Griess reagent. A two-step diazotization reaction occurrs, in which the nitrosating agent dinitrogen trioxide (N_2_O_3_), generated from the autoxidation of NO or from the acid-catalyzed formation of nitrous acid from nitrite, reacts with sulfanilamide to give a diazonium derivative, which is then coupled to N-(1-napthyl)ethylenediamine to form a colored diazo product that strongly absorbs at 540 nm [39].

**Figure 4:**
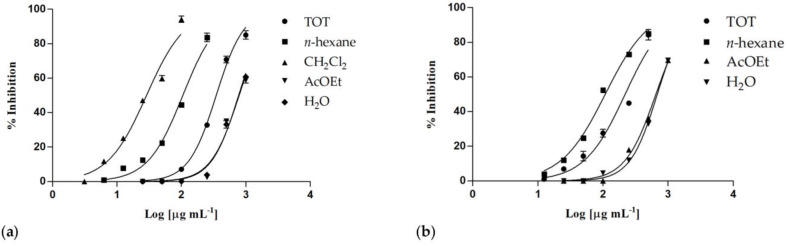
Nitric oxide production inhibition induced by (**a**) *Centranthus ruber* (L.) DC. and (**b**) *Tropaeolum majus* L. extracts and fractions. Data are expressed as mean ± S.E.M. (n = 4).

Both *C. ruber* and *T. majus* raw extracts showed concentration-dependent inhibitory properties (Figure 4), as well as almost all their fractions. 

The phytocomplex obtained from *T. majus* showed an inhibitory activity higher than *C. ruber* crude extract, with IC_50_ values equal to 219.4 ± 8.4 and 348.0 ± 8.3 μg/mL, respectively (Table 4). Although the samples from *T. majus* induced some cytotoxic effects on treated cells at the highest tested concentrations (IC_50_ = 613.4 ± 17.3 μg/mL), this cytotoxicity was very low at 250 μg/mL (4.1%), and it was not detectable at the lowest ones.

The most active sample was the dichloromethane fraction of *C. ruber* extract, with an IC_50_ value of 28.77 ± 1.2 μg/mL, a very interesting result if compared to the two utilized positive controls, the known anti-inflammatory drug indomethacin (IC_50_ = 58.0 ± 0.9 μg/mL) and the nitric oxide synthase inhibitor N^G^-nitro-L-arginine methyl ester (L-NAME, IC_50_ = 45.9 ± 0.5 μg/mL).

Good results were also observed for the *n*-hexane fractions from both extracts, with IC_50_ values equal to 105.4 ± 3.2 μg/mL (*C. ruber*) and 108.5 ± 2.5 μg/mL (*T. majus*).

In addition, for these fractions, some cytotoxic effects were observed at the highest concentrations, but any toxic effect was detected at the lowest ones, and a statistically significant difference was observed among the two IC_50_ values (*p* < 0.05).

The potential anti-inflammatory activity of *T. majus* extract was reported by Tran and coworkers, who described the inhibitory properties on the LPS-induced inflammatory response in human peripheral blood mononuclear cells [36]. Moreover, the aqueous and hydroethanolic extracts of *T. majus* were demonstrated to exert radical scavenging activity against reactive nitrogen species, NO^•^, and to inhibit the activity of COX1 by approximately 60% [30].

### 2.5. Anti-Denaturation Effects

**Figure 5:**
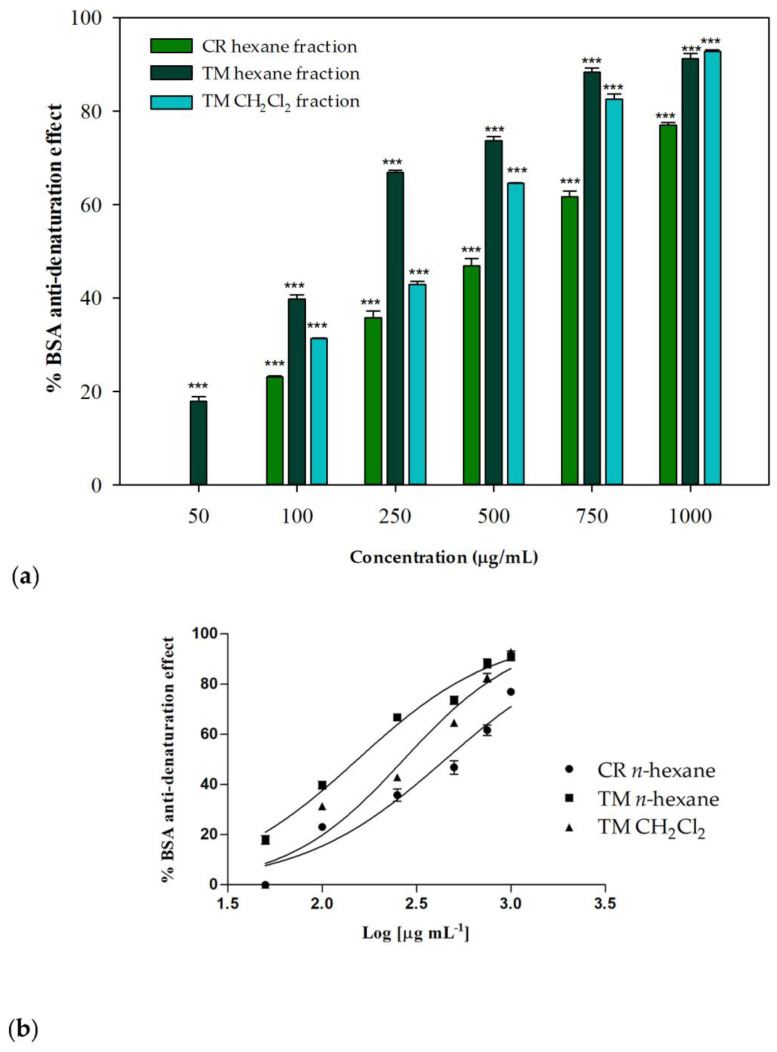
(**a**) Inhibition of BSA heat-induced denaturation. Data were expressed as mean ± S.E.M. (n = 3). Diclofenac sodium was used as a positive control. CR: *C. ruber* (L.) DC.; TM: *T. majus* L. Mean values of samples showing significant difference compared to the control (untreated 3.5% BSA water solution) were denoted with *** *p* < 0.001 (Dunnett’s multiple comparison test). (**b**) Nonlinear regression analyses.

The anti-denaturation effects of the investigated extracts and their fractions were verified in vitro using bovine serum albumin (BSA) as a protein model. Each sample was tested at different concentrations (range 50–1000 µg/mL) and incubated in the presence of a 3.5% BSA water solution at 37 °C and then heated at 71 °C for 1 min, in order to evaluate the inhibitory properties on the protein heat-induced denaturation. In the control group, water was used instead of samples. The untreated control represents the 100% BSA lysis. The well-known nonsteroidal anti-inflammatory drug (NSAID) diclofenac sodium was used as a positive control. Some of the investigated samples showed interesting concentration-dependent anti-denaturation effects on heat-treated BSA. As regards the whole phytocomplexes obtained from the two investigated species, only a moderate or low biological activity was observed: at the highest tested concentration (1000 µg/mL), *C. ruber* and *T. majus* raw extracts were able to induce about the 47% inihibition and the 27% of protein denaturation, respectively. On the contrary, their apolar fractions, namely the *n*-hexane sample from *C. ruber* extract and the *n*-hexane and dichloromethane fractions of *T. majus* crude extract, were effective in protecting the protein from heat-induced denaturation (Figure 5).

At the highest tested concentration (1000 µg/mL), inhibition percentages greater then 90% were detected for *T. majus* active fractions, while a percentage equal to 77.01% was observed for the *C. ruber n*-hexane fraction. The *T. majus n*-hexane fraction was particularly active, being able to inhibit BSA denaturation by 73.69% at 500 µg/mL and by 66.87% at 250 µg/mL. At the lowest tested concentration, 50 µg/mL, this sample was the only one still effective, being able to significantly inhibit heat-induced denaturation of BSA compared to the control (17.95%, *p* < 0.001, Dunnett’s multiple comparison test).

The raw data were fitted through nonlinear regression in order to deduce the IC_50_ parameters. Figure 5b shows the nonlinear regression analyses performed using Graph-Pad Prism 5. An IC_50_ value of 450.1 ± 15.5 μg/mL was observed for the *n*-hexane fraction from *C. ruber* extract, while better results were obtained for *T. majus* fractions, with IC_50_ values equal to 154.0 ± 1.9 and 270.8 ± 2.3 μg/mL for the *n*-hexane and dichloromethane samples, respectively.

To the best of our knowledge, this is the first report about the in vitro inhibitory effects of *C. ruber* and *T. majus* extracts on protein denaturation. Arthritis is a major cause of disability and, consequently, it constitutes one of the most common chronic illness worldwide, including a number of disorders, among which osteoarthritis and rheumatoid arthritis are the most frequent forms [40]. The pharmacological management of arthritis is mostly aimed at reducing inflammation and pain and is based on the use of non-steroidal anti-inflammatory drugs (NSAIDs), oral glucocorticoids and the more recently introduced disease-modifying antirheumatic drugs (DMARDs) and biologic drugs, or immunosuppressive and cytotoxic drugs [41,42]. As, overall, these agents also cause a number of toxic side effects, the interest towards plant extracts and molecules of natural origin is increasing. As a consequence, the number of papers dealing with potential health benefits of botanicals and nutritional supplements has increased in recent years, and different plants species have been reported to show antiarthritic potential [43,44,45].

### 2.6. In Vitro Pancreatic Lipase Inhibition

Given the traditional culinary use of both investigated plant species, we also aimed to investigate their nutraceutical value through the evaluation of the potential health benefits in the management of obesity. Obesity is an increasing public health problem also associated with a number of diseases, such as type II diabetes, cardiovascular diseases and cancer, and also including osteoarthritis [46]. 

In order to evaluate the potential anti-obesity activity of investigated samples, the in vitro porcine pancreatic lipase inhibitory potential was verified by monitoring the hydrolysis of 4-nitrophenyl caprylate (NPC), used as a substrate, and the release the yellow chromogen *p*-nitrophenol. Orlistat was used as a positive control. This drug is an effective gastrointestinal lipase inhibitor able to prevent dietary fat absorption by 30%, inhibiting both pancreatic and gastric lipase [46]. 

**Figure 6:**
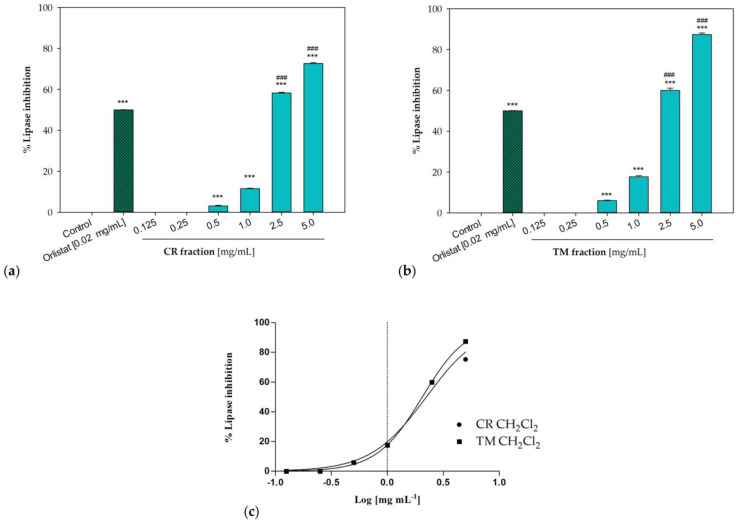
Pancreatic lipase inhibition induced by the dichloromethane fractions of (**a**) *C. ruber* (L.) DC. and (**b**) *T. majus* L. raw extract. (**c**) Non-linear regression curves. Data were expressed as mean ± S.E.M. (n = 3). Orlistat (0.02 mg/mL) was used as a positive control. CR: *C. ruber* (L.) DC.; TM: *T. majus* L. Significant difference versus control: *** *p* < 0.001; significant difference versus positive control: ### *p* < 0.001 (Dunnett’s multiple comparison test).

The dichloromethane fractions from both raw extracts were effective in inhibiting pancreatic lipase enzyme. Obtained results were consistent with a concentration-dependent inhibitory activity, with IC_50_ values equal to 2.23 ± 0.02 mg/mL for the sample from *C. ruber* and 2.05 ± 0.02 mg/mL for the *T. majus* fraction (Figure 6). At the highest tested concentration (5 mg/mL), inhibitory percentages equal to 72.65 ± 0.42% and 87.36 ± 0.73% were detected for *C. ruber* and *T. majus* samples, respectively, while the enzyme inhibition was equal to 58.26 ± 0.31% and 60.03 ± 1.06% at 2.5 mg/mL.

Potential health benefits in the prevention and treatment of obesity have already been hypothesized by Kim and coworkers, who reported the anti-adipogenic effects of *T. majus* flower ethanolic extract on 3T3-L1 cells [47]. The authors demonstrated that the *T. majus* sample was able to inhibit the lipid accumulation and to regulate adipocyte differentiation through the reduction in the expression of PPARG (peroxisome proliferator-activated receptor-γ), CEBPA (CCAAT/enhancer-binding protein-alpha) and SREBF1 (sterol regulatory element-binding transcription factor 1).

Moreover, some phytochemicals identified in these two active fractions demonstrated a very interesting lipase inhibitory potential. Cinnamic acid, detected in both samples, interestingly showed in vivo anti-obesity activity in high fat diet-fed rats [48]. The monoterpene lactone loliolide was demonstrated to significantly reduce the intracellular lipid accumulation in 3T3-L1 adipocytes [49]. 

## 3. Materials and Methods

### 3.1. Plant Material and Extraction Procedure

The aerial parts from wild *Centranthus ruber* and *Tropaeolum majus* were collected in Calabria (southern Italy) in June and May, respectively (leg. F. Conforti, det. F. Conforti). Voucher specimens are deposited in the Herbarium of the University of Calabria under the registration numbers CLU- 26241 (for *C. ruber)* and CLU- 26244 (for *T. majus*). Dried plant material was extracted with methanol (analytical grade, VWR International, Milan, Italy) at room temperature through maceration (plant to solvent ratio 1:10 g/mL, 48 h × 3 extractions). The obtained solutions were then filtered using qualitative filter paper with particle retention 10–20 μm (VWR International, Leuven, Belgium) and dried using a rotary evaporator IKA^®^ RV 10 (VWR International, Milan, Italy). 

A fraction of each raw extract was then suspended in methanol:water, 9:1 and extracted with *n*-hexane. The residue was then suspended in distilled water and extracted successively with dichloromethane and ethyl acetate (analytical grade, VWR International, Milan, Italy). 

### 3.2. GC-MS Analyses

GC-MS analyses were performed using a gas chromatograph (Hewlett-Packard, Model 6890, Milan, Italy) coupled with a selective mass detector (Hewlett Packard Model 5973, Milan, Italy). A 10 µL GC microsyringe (Hamilton, Bonaduz, Switzerland) was used to inject samples (1 µL), and the instrument was equipped with a SE-30 (100% dimethylpolysiloxane) capillary column (30 m × 0.25 mm, 0.25 μm film thickness). The carrier gas used was helium, and the linear velocity was equal to 0.00167 cm/s. A programmed temperature scheme with an increasing temperature from 60 °C to 280 °C and a rate of 16°/min was used. Column inlet was set at 250 °C. The other settings were: ion source, 70 eV; ion source temperature, 230 °C; electron current, 34.6 μA; vacuum 10−5 torr. Mass spectra were acquired over a 40–800 amu range at 1 scan/s. The identification of compounds was allowed by the comparison of their retention index (RI) with those in the literature, the comparison of mass spectra with the Wiley Mass Spectral Database of the GC–MS apparatus and also, for some compounds, the comparison with available standards (Sigma-Aldrich, Milan, Italy), as specified in Table 1 and Table 2 [50].

### 3.3. Quantification of Total phenolic and Flavonoid Content

Total phenolic content was estimated using the Folin–Ciocalteu method [51,52], in which phenols are oxidized by a mixture of tungstate and molybdate in a basic medium, causing the formation of colored molybdenum ions [53]. A 2 mg/mL solution of each raw extract dissolved in a solution of acetone/methanol/water/acetic acid 40:40:20:0.1 (analytical grade, VWR International, Milan, Italy) was incubated for 1 h at 60 °C. Then, 1 mL of Folin–Ciocalteu’s reagent (Sigma-Aldrich, Milan, Italy) and 1 mL of 7.5% sodium carbonate were added to 200 µL of each sample. Next, 2 h later, absorbance was measured at 726 nm with a UV-Vis spectrophotometer (Jenway 6300, Cole Parmer, UK). Data were interpolated with a chlorogenic acid standard calibration curve, and results were expressed as mg of chlorogenic acid equivalents per g of extract. 

Total flavonoid content was evaluated using the aluminum–chloride method [54]. A 2 mg/mL solution of *C. ruber* and *T. majus* extracts prepared in 80% ethanol (1 mL, (analytical grade, VWR International, Milan, Italy) was added to AlCl_3_ reagent (1 mL, (Sigma-Aldrich, Milan, Italy). Solutions were allowed to stay at room temperature for 15 min in the dark, and absorbance was then measured at 430 nm. Data were interpolated with a quercetin standard calibration curve; results were reported as mg of standard equivalent per g of extract. 

### 3.4. HPTLC Analyses

A CAMAG semi-automated high-performance thin layer chromatography (HPTLC) system including a Linomat 5 sample applicator connected to a TLC Visualizer and controlled with wincats planar chromatography software was utilized (CAMAG, Muttenz, Switzerland).

The ethyl acetate fractions from both extracts were analyzed by co-chromatography with the reference compounds. Samples were firtsly dissolved in methanol to a final concentration of 50 mg/mL. Samples (1 µL), together with standard reference compounds (3 mg/mL, 1 µL, Sigma-Aldrich, Milan, Italy), were spray-applied with a micro-syringe on normal phase silica gel glass plates (20 × 10 cm, silica 2–10 µm, Merck, Germany). 

Operating conditions were as follows: band width 8 mm; distance from bottom 1.5 mm; solvent front position 9 mm; syringe delivery speed 150 nL/s [55].

Plates were developed using the mobil phase ethyl acetate/dichloromethane/acetic acid/formic acid/water (100:25:10:10:11, v/v/v/v/v). Natural Product Reagent (NPR, 1 g diphenylborinic acid aminoethyl ester in 200 mL AcOEt) and anysaldehyde (1.5 mL *p*-anisaldehyde, 2.5 mL sulfuric acid, 1 mL acetic acid in 37 mL ethanol) were utilized for post-chromatographic derivatization. Reagents were obtained from Sigma-Aldrich (Milan, Italy). Plates were dipped into freshly prepared reagents and then dried at 100 °C for 5 min. 

Plates were inspected under UV light (254 and 366 nm) and white light (upper and lower) both before and after derivatization by means of the CAMAG TLC visualizer.

Band stability was verified examining the resolved peaks at intervals of 12, 24 and 48 h. Repeatability was determined by running three analyses. RF values for the main identified compounds varied by less than 0.02%.

### 3.5. In Vitro Methods for Antioxidant Activity Assessment

The radical scavenging activity of *C. ruber* and *T. majus* extracts and their fractions was evaluated using the well-established in vitro DPPH test [56]. Briefly, 200 µL of each sample solution (concentrations ranging from 5 to 1000 µg/mL in MeOH) was added to 800 µL of 2,2-diphenyl-1-picrylhidrazyl (DPPH, 0.1 mM, Sigma-Aldrich, Milan, Italy). The absorbance was measured at 517 nm 30 min later using a UV-Vis spectrophotometer (Jenway 6300, Cole Parmer, UK). Ascorbic acid (Sigma-Aldrich, Milan, Italy) was used as the positive control. 

The antioxidant activity was also assessed by means of the β-carotene bleaching test [50]. This colorimetric method is based on the measurement of the decay of the absorption at 470 nm linked to β-carotene bleaching caused by free radicals generated by the heat induced oxidation of linoleic acid [53]. Briefly, a β-carotene-linoleic acid emulsion was prepared by adding 100% Tween 20 (0.2 mL) to a 0.2 mg/mL β-carotene chloroform solution (1 mL) and to linoleic acid (0.02 mL, Sigma-Aldrich, Milan, Italy) and by successively removing the solvent and adding distilled water (100 mL). Then, 5 mL of such an emulsion were added to 0.2 mL of each sample (concentrations ranging from 0.25 to 100 µg/mL) and placed in a water bath at 45 °C. Propyl gallate (Sigma-Aldrich, Milan, Italy) was used in the positive control group. The oxidation was monitored by measuring the absorbance at 470 nm over a 60 min period, namely, at the initial time and after 30 and 60 min. 

### 3.6. In Vitro Evaluation of the Inhibitory Properties on Nitric Oxide (NO) Production

The inhibitory effects on NO production were verified in vitro on lipopolysaccharide (LPS)-stimulated RAW 264.7 macrophages (ATCC, UK, No. TIB-71) as previously described [57]. Cells were cultured in Dulbecco’s modified Eagle’s medium (DMEM, Sigma-Aldrich, Milan, Italy) supplemented with L-glutamine, fetal bovine serum and a solution of penicillin and streptomycin (1%, 10% and 1%, respectively, Sigma-Aldrich, Milan, Italy). For the experiments, cells were seeded into 96 well microtiter plates (1 × 10^5^ cells/well) after having been removed from a culture flask by scraping. Then, 24 hours later, plates were treated with samples (concentrations ranging from 1000 to 3 µg/mL, final ratio of DMSO to medium 0.5% v/v) and stimulated with 1 µg/mL LPS. 

The presence of nitrite was measured after a further 24 h with the Griess reagent (Sigma-Aldrich, Milan, Italy). To this end, 100 µL of reagent were added to 100 µL of the cell culture supernatant of each well, and absorbance was measured at 550 nm using a microplate reader (Stat fax 3200, Awareness Technology Inc., Palm City, FL, USA). The known anti-inflammatory drug indomethacin and the NO synthase inhibitor L-NAME were used as positive controls (Sigma-Aldrich, Milan, Italy).

The cytotoxic potential of samples on treated cells was assessed by means of the well-known MTT test [55]. Briefly, after the removal of cell supernatants, a 0.5% MTT tetrazolium salt solution in PBS (phosphate-buffered saline, 100 µL, Sigma-Aldrich, Milan, Italy) was added to each well. After 4 h of incubation, the solution was removed and DMSO (100 µL) was added. The absorbance was measured at 550 nm. 

### 3.7. In Vitro Anti-Denaturation Effects

The anti-denaturation effects of investigated ornamental plants were evaluated on heat-treated bovine serum albumine, chosen as the protein model, as previously reported [16]. An amount of 0.1 mL of each sample (concentration range 50–1000 µg/mL in DMSO) was added to 2.40 mL of a 3.5% BSA water solution (Sigma-Aldrich, Milan, Italy). Water was utilized in the untreated control group and diclofenac sodium was used as the positive control. A product control group without BSA was also utilized. The pH was adjusted at 6.3 with HCl (1 N). After 20 min of incubation at 37 °C, the obtained solutions were heated at 71 °C for 1 min. After cooling, samples were diluted with 2.5 mL of a phosphate buffered saline (pH 6.3), prepared as follows: NaCl (8 g), KCl (0.2 g), Na_2_HPO_4_ (1.44 g) and KH_2_PO_4_ (0.24 g), obtained from Sigma-Aldrich (Milan, Italy), were dissolved in 800 mL of distilled water. The pH was adjusted at 6.3 using 1 N HCl and distilled water was added in order to reach a final volume of 1000 mL.

The turbidity of the obtained solutions was measured spectrophotometrically at 660 nm using a UV-Vis spectrophotometer (Jenway 6300, Cole Parmer, UK).

### 3.8. Measurement of Pancreatic Lipase Inhibition

The potential anti-obesity effects of investigated extracts and fractions were assessed in vitro, with the pancreatic lipase inhibitory assay, as previously described [58]. A 1 mg/mL water solution of type II crude porcine pancreatic lipase (25 µL, Sigma-Aldrich, Milan, Italy) was mixed with 5 mM NPC (25 µL, Sigma-Aldrich, Milan, Italy), tris-HCl buffer (pH 8.5, 1 mL, Sigma-Aldrich, Milan, Italy) and *C. ruber* and *T. majus* extracts and fractions (25 µL, concentration range 0.125–5 mg/mL). The obtained solutions were incubated at 37 °C for 25 minutes. The absorbance was measured at 412 nm using a Jenway 6300 UV-Vis spectrophotometer (Cole Parmer, UK). Orlistat was used as a potivite control.

### 3.9. Statistical analysis

The experiments were run in triplicate, with the exception of the assays involving cell cultures, for which four replicates were performed. Data were expressed as mean ± SEM. In order to check the normality of data and the homogeneity of variance, the Pearson’s K2 test and the Levene’s test were used. Non-linear regression analyses were performed using Graph-Pad Prism 5 (Graph Pad Software Inc., San Diego, CA, USA). Statistical differences were assessed through one-way ANOVA (*p* < 0.05, Sigma Stat Software, Jandel Scientific Software, San Rafael, CA, USA). Pairwise post-hoc comparisons were performed using the Bonferroni post-hoc test; Dunnett’s multiple comparison test was performed to compare the treated groups and the control.

The MetaboAnalyst software v. 5.0 was used to perform the Principal Component Analysis (PCA) (http://www.metaboanalyst.ca, accessed on 6 November 2022). Before data analysis, an integrity check was performed. Zero values were replaced with small values (1/5 of the min positive value for each variable), and data were normalized using Pareto-scaling. 

## 4. Conclusions

Different botanicals have been demonstrated to play a potential role in the treatment of arthritis [59,60] and obesity [61]. Amidst the wide diversity of plant species, ornamental plants also contribute to the huge numbers of isolated bioactive phytochemicals that have been identified up to now [4]. 

Starting from these considerations, the scope of this study was to investigate two species, *Centranthus ruber* (L.) DC. and *Tropaeolum majus* L., mainly used as ornamental plants but also traditionally consumed as food and also used in popular medicine. 

Different phytochemicals were detected with GC-MS analyses. Our results contribute to the knowledge of the phytochemical profile of these two plant species, mainly studied in literature for their phenolic profile. Here, the presence of different classes of more nonpolar compounds was described, e.g., terpenoids, fatty acids and phytosterols.

Previous studies in literature evidenced the anti-inflammatory potential of *T. majus* extracts [30,36]. Taking into account these reports, one of the aims of our study was to investigate the potential antiarthritic activity of both investigated plant species. The *n*-hexane and dichloromethane fractions from *T. majus*, as well as the *n*-hexane fraction from *C. ruber*, demonstrated protective effects on heat-induced protein denaturation. Moreover, almost all the extracts and fractions demonstrated good radical scavenging activity and inhibitory effects on NO production. These last results were particularly interesting as, among a wide range of biological functions, NO has also been demonstrated to play a role in the progression of osteoarthritis. Increased NO levels, produced by iNOS, constitute a catabolic element in the activation of TNFα and IL-1β, being related to cartilage chondrocyte apoptosis [35].

Moreover, given the traditional culinary use of *C. ruber* and *T. majus*, we aimed to assess their nutraceutical value, and our study evidenced good inhibitory properties on the digestive enzyme pancreatic lipase. This enzyme plays a key role for the efficient digestion of dietary fats, and the inhibition of digestive enzymes is considered to be one of the most promising strategies for weight loss and weight management [62]. The dichloromethane fractions from both raw extracts showed interesting in vitro inhibitory properties. These results are also particularly interesting considering that a link between obesity and osteoarthritis has been established, even if the exact mechanism of the relationship has not yet been fully understood [63]. 

To the best of our knowledge, this is the first report concerning the in vitro inhibitory properties on protein denaturation and on pancreatic lipase enzyme for these plants. The obtained results suggest that *C. ruber* and *T. majus* could be a potential source of antiarthritic and anti-obesity agents. Future studies could be useful to optimize the solvent partitioning method and to continue investigating the interesting biological properties of these species.

## Figures and Tables

**Table 1:** Phytochemical profile of the *n*-hexane fractions of *Centranthus ruber* (L.) DC. and *Tropaeolum majus* L. extracts.

No.	Compound	RI ^1^	RAP ^2^		ID ^3^
			*C. ruber* (L.) DC.	*T. majus* L.	
	Fatty Acids				
1	Isolauric acid	1401	0.10 ± 0.01	-	a,b
2	Lauric acid	1590	0.12 ± 0.01	0.50 ± 0.04	a,b
3	Azelaic acid	1665	0.10 ± 0.01	-	a,b
4	Myristic acid	1798	0.21 ± 0.02	0.22 ± 0.02	a,b
5	Pentadecanoic acid	1866	0.21 ± 0.02	0.20 ± 0.02	a,b
6	7,10,13-Hexadecatrienoic acid	1949	-	0.20 ± 0.02	a,b
7	Palmitic acid	1970	6.87 ± 0.23	5.08 ± 0.20	a,b
8	Margaric acid	2050	-	0.10 ± 0.01	a,b
9	α-Linolenic acid	2158	-	0.29 ± 0.01	a,b
10	Linoleic acid	2169	-	0.51 ± 0.04	a,b
11	9-Octadecenoic acid	2183	-	1.51 ± 0.05	a,b
12	Stearic acid	2199	1.02 ± 0.03	1.00 ± 0.08	a,b
13	Arachidic acid	2350	-	0.41 ± 0.03	a,b
14	Heneicosanoic acid	2459	-	0.10 ± 0.01	a,b
15	Behenic acid	2560	2.13 ± 0.06	0.39 ± 0.03	a,b
16	Tricosylic acid	2662	0.51 ± 0.03	-	a,b
	Terpenes				
17	2,6-Di-tert-butyl-1,4-benzoquinone	1452	0.20 ± 0.02	-	a,b,c
18	Dihydroactinidiolide	1573	0.41 ± 0.03	0.10 ± 0.01	a,b
19	Neophytadiene	1777	-	0.40 ± 0.02	a,b,c
20	Phytone	1830	2.80 ± 0.05	0.60 ± 0.04	a,b
	Phytosterols				
21	Campesterol	3107	-	1.20 ± 0.04	a,b
22	Stigmasterol	3150	-	0.10 ± 0.01	a,b
23	β-Sitosterol	3182	2.10 ± 0.04	-	a,b
24	Tremulone	3438	0.94 ± 0.06	-	a,b
	Others				
25	β-Tocopherol	3070	-	0.30 ± 0.02	a,b

^1^ Retention indices on GC-MS column. ^2^ Relative area percentage (peak area relative to total TIC peak area %). ^3^ ID, Identification method as indicated by the following: (a) comparison of retention index; (b) comparison of mass spectrum with the MS libraries; (c) co-injection with authentic standard compound. Each value is the mean ± S.D. of three independent measurements.

**Table 2:** Chemical constituents identified in the dichloromethane fractions of *Centranthus ruber* (L.) DC. and *Tropaeolum majus* L. extracts.

No.	Compound	RI ^1^	RAP ^2^		ID ^3^
			*C. ruber* (L.) DC.	*T. majus* L.	
26	3-Cresol	1046	0.10 ± 0.01	-	a,b
27	Benzoic acid	1158	1.10 ± 0.02	-	a,b
28	Ethylmethylmaleimide	1230	0.51 ± 0.03	0.10 ± 0.01	a,b
29	Cinnamic acid	1421	0.20 ± 0.02	0.10 ± 0.01	a,b
30	Isoeugenol	1436	0.20 ± 0.01	0.10 ± 0.01	a,b,c
31	2,6-Bis(tert-butyl)phenol	1454	0.40 ± 0.03	-	a,b
32	Mandelic acid	1480	-	6.63 ± 0.10	a,b
33	Vanillic acid	1560	-	0.10 ± 0.01	a,b
34	Loliolide	1696	-	2.21 ± 0.04	a,b

^1^ Retention indices on GC-MS column. ^2^ Relative area percentage (peak area relative to total TIC peak area %). ^3^ ID, Identification method as indicated by the following: (a) comparison of retention index; (b) comparison of mass spectrum with the MS libraries; (c) co-injection with authentic standard compound. Each value is the mean ± S.D. of three independent measurements.

**Table 3:** Antioxidant activity of *C. ruber* (L.) DC. and *T. majus* L. extracts and fractions.

Species	Sample	IC_50_ (μg/mL)		
		DPPH Test	β-Carotene Bleaching Test	
			30 min	60 min
*Centranthus ruber* (L.) DC.	raw extract	79.86 ± 1.27 ^d^	38.15 ± 1.34 ^c^	62.58 ± 2.51 ^e^
	*n*-hexane fraction	n.a.	n.a.	n.a.
	CH_2_Cl_2_ fraction	119.50 ± 1.44 ^e^	n.a.	n.a.
	AcOEt fraction	84.22 ± 0.48 ^d^	28.17 ± 0.49 ^b^	55.61 ± 0.38 ^d^
	H_2_O fraction	151.8 ± 1.75 ^f^	49.48 ± 1.20 ^d^	79.59 ± 2.23 ^g^
*Tropaeolum majus* L.	raw extract	53.34 ± 0.34 ^c^	39.57 ± 0.49 ^c^	70.53 ± 1.54 ^f^
	*n*-hexane fraction	n.a.	n.a.	n.a.
	CH_2_Cl_2_ fraction	53.72 ± 0.52 ^c^	49.00 ± 0.93 ^d^	n.a.
	AcOEt fraction	14.08 ± 0.29 ^b^	41.68 ± 1.38 ^c^	63.41 ± 0.17 ^e^
	H_2_O fraction	77.55 ± 2.81 ^d^	n.a.	n.a.
Ascorbic acid ^1^		2.00 ± 0.01 ^a^	-	-
Propyl gallate ^1^		-	1.00 ± 0.02 ^a^	1.00 ± 0.02 ^a^

Data are expressed as mean ± SEM (n = 3). n.a., not active. Different superscript letters (a–f), along column for DPPH test and between 30 and 60 min columns for β-carotene bleaching test, indicate statistically significant differences with *p* < 0.05 (Bonferroni post-hoc test). ^1^ Positive controls.

**Table 4:** In vitro inhibitory properties on NO production of *C. ruber* (L.) DC. and *T. majus* L. extracts and fractions.

Species	Sample	IC_50_ (μg/mL)	
		NO Inhibition	Cytotoxicity
*Centranthus ruber* (L.) DC.	raw extract	348.0 ± 8.3 ^d^	n.a.
	*n*-hexane fraction	105.4 ± 3.2 ^b^	300.2 ± 3.7 ^d^
	CH_2_Cl_2_ fraction	28.77 ± 1.2 ^a^	221.7 ± 2.1 ^c^
	AcOEt fraction	789.1 ± 24.3 ^g^	n.a.
	H_2_O fraction	775.8 ± 21.6 ^g^	n.a.
*Tropaeolum majus* L.	raw extract	219.4 ± 8.4 ^c^	613.4 ± 17.3 ^e^
	*n*-hexane fraction	108.5 ± 2.5 ^b^	225.3 ± 5.3 ^c^
	CH_2_Cl_2_ fraction	n.a.	711.6 ± 17.3 ^f^
	AcOEt fraction	651.2 ± 15.8 ^e,f^	744.1 ± 6.8 ^f,g^
	H_2_O fraction	683.8 ± 15.6 ^f^	n.a.
Indomethacin ^1^		58.0 ± 0.9 b ^a,b^	-
L-NAME ^1^		45.9 ± 0.5 ^a,b^	-

Data are expressed as mean ± SEM (n = 4). n.a., not active. Different letters indicate statistically significant differences with *p* < 0.05 (Bonferroni post-hoc test). ^1^ Positive controls.

## Data Availability

The data presented in this study are available in the article.

## Sample Availability

Samples of the compounds are not available from the authors.
